# Imaging-Based Subtypes of Pancreatic Ductal Adenocarcinoma Exhibit Differential Growth and Metabolic Patterns in the Pre-Diagnostic Period: Implications for Early Detection

**DOI:** 10.3389/fonc.2020.596931

**Published:** 2020-12-02

**Authors:** Mohamed Zaid, Dalia Elganainy, Prashant Dogra, Annie Dai, Lauren Widmann, Pearl Fernandes, Zhihui Wang, Maria J. Pelaez, Javier R. Ramirez, Aatur D. Singhi, Anil K. Dasyam, Randall E. Brand, Walter G. Park, Syed Rahmanuddin, Michael H. Rosenthal, Brian M. Wolpin, Natalia Khalaf, Ajay Goel, Daniel D. Von Hoff, Eric P. Tamm, Anirban Maitra, Vittorio Cristini, Eugene J. Koay

**Affiliations:** ^1^ Department of Radiation Oncology, The University of Texas MD Anderson Cancer Center, Houston, TX, United States; ^2^ Mathematics in Medicine Program, Houston Methodist Research Institute, Houston, TX, United States; ^3^ Department of Pathology, University of Pittsburgh Medical Center, Pittsburgh, PA, United States; ^4^ Department of Radiology, University of Pittsburgh, Pittsburgh, PA, United States; ^5^ Department of Medicine, University of Pittsburgh, Pittsburgh, PA, United States; ^6^ Department of Medicine, Stanford University, Stanford, CA, United States; ^7^ Department of Radiology, City of Hope, Duarte, CA, United States; ^8^ Department of Radiology, Dana Farber Cancer Institute, Boston, MA, United States; ^9^ Department of Medical Oncology, Dana Farber Cancer Institute, Boston, MA, United States; ^10^ Section of Gastroenterology and Hepatology, Department of Medicine, Baylor College of Medicine, Houston, TX, United States; ^11^ Department of Molecular Diagnostics and Experimental Therapeutics, City of Hope, Duarte, CA, United States; ^12^ Molecular Medicine, Translational Genomics Research Institute, Phoenix, AZ, United States; ^13^ Department of Abdominal Imaging, The University of Texas MD Anderson Cancer Center, Houston, TX, United States; ^14^ Department of Pathology, The University of Texas MD Anderson Cancer Center, Houston, TX, United States

**Keywords:** pancreatic cancer, early detection, computed tomography, mathematical modeling, tumor metabolism

## Abstract

**Background:**

Previously, we characterized subtypes of pancreatic ductal adenocarcinoma (PDAC) on computed-tomography (CT) scans, whereby conspicuous (high delta) PDAC tumors are more likely to have aggressive biology and poorer clinical outcomes compared to inconspicuous (low delta) tumors. Here, we hypothesized that these imaging-based subtypes would exhibit different growth-rates and distinctive metabolic effects in the period prior to PDAC diagnosis.

**Materials and methods:**

Retrospectively, we evaluated 55 patients who developed PDAC as a second primary cancer and underwent serial pre-diagnostic (T0) and diagnostic (T1) CT-scans. We scored the PDAC tumors into high and low delta on T1 and, serially, obtained the biaxial measurements of the pancreatic lesions (T0-T1). We used the Gompertz-function to model the growth-kinetics and estimate the tumor growth-rate constant (α) which was used for tumor binary classification, followed by cross-validation of the classifier accuracy. We used maximum-likelihood estimation to estimate initiation-time from a single cell (10^-6^ mm^3^) to a 10 mm^3^ tumor mass. Finally, we serially quantified the subcutaneous-abdominal-fat (SAF), visceral-abdominal-fat (VAF), and muscles volumes (cm^3^) on CT-scans, and recorded the change in blood glucose (BG) levels. T-test, likelihood-ratio, Cox proportional-hazards, and Kaplan-Meier were used for statistical analysis and p-value <0.05 was considered significant.

**Results:**

Compared to high delta tumors, low delta tumors had significantly slower average growth-rate constants (0.024 month^−1^ vs. 0.088 month^−1^, p<0.0001) and longer average initiation-times (14 years vs. 5 years, p<0.0001). α demonstrated high accuracy (area under the curve (AUC)=0.85) in classifying the tumors into high and low delta, with an optimal cut-off of 0.034 month^−1^. Leave-one-out-cross-validation showed 80% accuracy in predicting the delta-class (AUC=0.84). High delta tumors exhibited accelerated SAF, VAF, and muscle wasting (p <0.001), and BG disturbance (p<0.01) compared to low delta tumors. Patients with low delta tumors had better PDAC-specific progression-free survival (log-rank, p<0.0001), earlier stage tumors (p=0.005), and higher likelihood to receive resection after PDAC diagnosis (p=0.008), compared to those with high delta tumors.

**Conclusion:**

Imaging-based subtypes of PDAC exhibit distinct growth, metabolic, and clinical profiles during the pre-diagnostic period. Our results suggest that heterogeneous disease biology may be an important consideration in early detection strategies for PDAC.

## Introduction

Pancreatic ductal adenocarcinoma (PDAC) is the fourth most common cancer in the United States, with 57,600 new cases and 47,050 deaths projected annually (1). More than 80% of the new cases are either at regional or distant spread stage by the time of initial diagnosis, and without breakthroughs in therapeutics and early detection strategies, PDAC will become the second leading cause of cancer-related deaths in the US by 2030 (2, 3). Compared to other cancers, efficient imaging-based screening methods for PDAC are lacking (4, 5). While significant efforts have turned to defining high risk cohorts for screening efforts, most cases of early PDAC diagnosis are incidental findings on computed tomography (CT) or magnetic resonance (MR) scans that are performed for reasons other than the suspicion of PDAC (6, 7). Even in high risk cohorts, metastatic PDAC can develop while a subject is on surveillance (7). This highlights the need to identify ways to personalize screening strategies based on disease biology.

Multiple groups have recognized that the pre-diagnostic period for PDAC exhibits measurable changes that have given new insight into the systemic effects of the disease before it is clinically detected and diagnosed. For example, using pre-diagnostic images and blood tests, investigators showed that the emergence of PDAC is associated with muscle and fat wasting and changes in the glucose, protein, and lipid profiles (8–10). Large cohort studies and hospital systems have represented the main sources of data to date. Another source of patients in whom a pre-diagnostic period for PDAC could be studied is those who develop PDAC as a second primary malignancy. Second primary cancers constitute 16% of the newly diagnosed cancers in the United States, and second primary pancreatic cancer represents 6.3% of all new diagnosed pancreatic cancers, with a median interval of 8.4 years from the prior cancer (11–14). Many of these cancers are diagnosed by serial follow up scans performed for the purpose of management or surveillance of the first primary cancer. These scans can offer a unique opportunity to study the evolutionary nature of PDAC tumors. To this end, the application of physiologically-relevant mathematical models that can utilize serial scans, clinical and biological data to model tumor growth (15–18), and predict disease prognosis may help in evaluating screening strategies and achieving the goal of personalized approaches based on disease biology.

It is known that PDAC is a heterogeneous disease, and multiple methods have been proposed to classify the disease. Previously, we identified imaging-based subtypes of PDAC (19). We showed that qualitative and quantitative scoring of the change in enhancement on CT-scans at the interface between PDAC tumors and parenchyma (delta) is biologically and clinically relevant, whereby tumors with a conspicuous border (high delta) on CT show more aggressive mesenchymal biology, are more likely to have multiple common pathway mutations, and are associated with poor clinical outcomes, when compared to those with an inconspicuous border (low delta) on CT (19–22).

In this study, we hypothesized that high and low delta tumors have different growth kinetics and utilized image-guided mathematical modeling to characterize the differences in growth patterns. We used measurements derived from serial pre-diagnostic and diagnostic CT-scans of patients who developed PDAC as a second primary cancer to inform a phenomenological mathematical model of tumor growth and estimate relevant growth parameters. The modeling results were utilized to perform binary classification of tumors into high delta and low delta, solely based on their growth kinetics differences. Lastly, we tested the hypothesis that these imaging-based subtypes have different rates of soft tissue wasting, changes in blood glucose (BG) levels, and clinical outcomes.

## Materials and Methods

### Patients

This study was approved by the Institutional Review Board (PA14-0646) at The University of Texas MD Anderson Cancer Center (MDACC). Retrospectively, we evaluated a cohort of 55 patients who developed pathologically proven PDAC as a second primary cancer between the years 2003 and 2019. All patients had undergone at least one pre-diagnostic CT-scan (T0) as a follow up for their primary cancer that showed a pancreatic lesion and a diagnostic (pre-therapy) pancreatic protocol CT-scan (T1) for PDAC (**Supplementary Table S1**).

### CT Acquisition

Due to the retrospective nature of the study the pre-diagnostic CT-scan(s) (T0) acquisition protocols varied. However, for all patients there was at least one contrast-enhanced T0 CT-scan that was used for tumor measurement. Diagnostic CT- scans (T1) were acquired using pancreatic-protocol, which is a diagnostic test for patients with PDAC, where iodine-based contrast is injected intravenously (23). Fixed-time delay technique was used in scans obtained before 2006 (n=4), which consisted of a non-contrast (NC), an arterial (AR) phase (40 s after starting contrast infusion) and a portovenous (PV) phase (65–70 s after starting contrast infusion). Scans obtained after 2006 used a bolus tracking technique (n=51), whereby a value of 100 HU in the aorta triggers the countdown to start the AR phase scan, followed by the PV phase. The slice thickness for post contrast scans ranged between 2.5 mm and 3 mm.

### CT-Analysis: Delta Scoring and Tumor Measurement

Qualitatively, we scored the imaging-based subtypes of PDAC tumors on (T1) diagnostic CT-scans based on conspicuity and shape into low and high delta groups using previously published criteria (19). Then, we measured pancreatic lesions on the contrast-enhanced CT images at the following time points: I) T0(s): when a pancreatic abnormality (lesion) was first radiologically visible and every follow-up scan until before PDAC diagnosis was made; II) T1: when the PDAC diagnosis was radiologically established. Measurement included the long and short axes of the lesions, which were geometrically averaged to obtain a reasonable approximation of the mean lesion diameter (d). Then, by approximating the lesion as a sphere, we obtained the lesion volume (cm^3^). This tumor volume estimation method is verified to have a high correlation with the actual 3D volume (24).

### Empirical Mathematical Modeling

To appropriately model the growth kinetics of the tumors, we used the Gompertz function given by:

$$X(t)=Ke^{\ln\left(\frac{X_{0}}{K}\right)e^{-\alpha t}}$$

where, *X*(*t*) refers to the volume of the tumor at a given time *t*; *K* is the tumor carrying capacity of the host, i.e. maximum tumor volume that can be achieved in the body under the limitations of nutrient availability (value fixed at 180 cm^3^, which is equivalent to a sphere of 7 cm dia.);*X*_o_ is the volume of the tumor at the first observation, which was assumed to be at time zero (T0) and obtained from the data; and α is the growth rate constant of the tumor.

The Gompertz function was fit to each patient’s longitudinal tumor size data to estimate the growth rate constant α. The fitted function was used for backward projection to estimate the time to grow (initiation time *T*) from a single cancerous cell (≈10^-6^ mm^3^) to a tumor of 10 mm^3^ size (10 million cells) using the following formula:

$$T=\left|-\frac{1}{\alpha }\ln\left(\frac{\ln(X(t_{0})/K)}{\ln(X_{0}/K)}\right)\right|-\left|-\frac{1}{\alpha }\ln\left(\frac{\ln(X(t_{1})/K)}{\ln(X_{0}/K)}\right)\right|$$

where, *X*(*t*_0_) and *X*(*t*_1_) are the volume of a single cell and diagnosable tumor mass, respectively (i.e., 10^-6^ mm^3^ and 10 mm^3^ in our calculations, respectively).

### Binary Classification and Cross-Validation

The entire data set (*n* = 55) was used to train the binary classifier (low versus high delta tumor types). A logistic regression model was fit between the predictor (growth rate constant α) and response (tumor type) variables, and a receiver operating characteristic (ROC) curve was computed. Accuracy of classification was obtained as the percentage of tumors correctly classified by the ‘discrimination threshold’ that was selected from the ROC curve to maximize the accuracy of classification.

Leave-one-out cross validation (LOOCV) technique was used to evaluate the predictive capability of the binary classifier. In this technique, *n*-1 training data sets were generated from the total *n* data points by iteratively removing one data point. Each training dataset was used to generate a ROC curve and select a discrimination threshold to classify the left-out test data point. The prediction results from all iterations were pooled to calculate the average accuracy of the classifier.

### Soft Tissue and Metabolic Profile Assessment

We quantified the change in subcutaneous abdominal fat (SAF), visceral abdominal fat (VAF) and muscle area on a single axial slice of each CT scan at the L2-L3 vertebral level. In brief, we imported the CT images to velocity AI software (Varian Medical Systems, Inc.) and used the provided semi-automated segmentation tool to contour and obtain the area on the single slice (25). We then calculated the volume (cm^3^) of SAF, VAF and muscle using knowledge of the CT slice thickness. This was serially done for T0(s) and T1.

To construct a temporal metabolic profile for these patients, we serially collected the blood glucose (BG) level from the electronic medical records of the patients starting from the date of diagnosis and up to 24 months prior to diagnosis.

### Inter- and Intra-Rater Variability

To evaluate inter-rater agreement, two trained researchers (DE and MZ), with 3 and 4 years of experience reviewing pancreatic cancer CT scans, respectively, performed serial biaxial measurement of the pancreatic lesions in randomly selected cases (30% of the studied cohort, 16 cases total). Then we calculated the lesion’s volume by geometrically averaging the biaxial measurement and approximating the lesion as a sphere.

To evaluate intra-rater agreement, repeated measurements were performed by one rater (MZ) for the same 16 cases (>2 weeks interval), and similarly calculated the volume of the lesions.

We used the intraclass correlation coefficient (ICC) test to evaluate inter-rater (two-way random effects, absolute agreement) and intra-rater (two-way mixed effects, absolute agreement) We reported the agreement rates according to the published guidelines.

### Statistical Analysis

Non-linear least squares regression using the “Levenberg-Marquardt” algorithm was performed to fit the Gompertz function to each patient’s tumor volume data. To evaluate the quality of model fits, Pearson’s correlation coefficient *R* was calculated between the observed data and model fitted predictions. Maximum likelihood estimation was performed to estimate the parameters of lognormal distribution to characterize the distribution of initiation times. The maximum likelihood estimates were then used to obtain the probability density function and cumulative density function (cumulative probability) of initiation times.

T-test and likelihood ratio were used for comparative numeric and categorical analysis, respectively between the groups. Cox proportional-hazards and Kaplan-Meier were used for overall survival (OS) and progression free survival (PFS) analyses. Statistical analyses were performed in MATLAB R2018a (MathWorks), JMP Pro 15 (SAS Institute), and Prism (GraphPad). All tests were two-tailed and p-value <0.05 was considered significant.

## Results

### Patient Population

Our patient population consisted of 33 males (60%) and 22 females (40%), the median age at the time of diagnosis for PDAC was 68 years (range = 50–87), the median time interval between the first and second cancer was 4.5 years (range = 0.1–42) and the median overall survival (OS) time after the PDAC diagnosis date was 22 months (range = 2–154). At time of diagnosis, 34 patients had stage I and II disease, 7 patients had stage III disease and 14 had stage IV disease. Ten patients underwent surgical resection for PDAC, of which four patients had stage I tumors, and six patients had stage II tumors according to the American Joint Committee on Cancer (AJCC) 8^th^ edition. Twenty-nine patients had high delta tumors, while 26 had low delta tumors. Patients’ demographics and clinical variables related to PDAC are shown in **Table 1**. Clinical variables related to the first primary cancer are shown in **Table 2** and **Supplementary Table S2**.

**Table 1:** Demographic and treatment characteristics.

Characteristic	No. (%)
**Age** (median-range)	68.3 [50-87]
**Sex**	
Female	22 (40)
Male	33 (60)
**Race**	
Caucasian	43 (72)
Black	4 (7)
Hispanic	8 (14)
**Surgery**	
Yes	10 (18)
No	45 (82)
**Pathological T stage**	
T1	1 (1)
T2	8 (8)
T3	1 (1)
T4	–
**Pathological N stage**	
N0	6(64)
N1	4 (36)
N2	0
**Clinical Stage**	
I	21 (38)
II	13 (24)
III	7 (13)
IV	14 (25)
**Surgical Stage (AJCC 8^th^)**	
IA	1
IB	3
IIA	2
IIB	4
III	–
IV	–
**Surgical margin**	
Negative (R0)	09 (90)
Positive (R1)	1 (10)
**Chemotherapy**	
Yes	42 (76)
No	13 (24)
**Radiotherapy**	
Yes	11 (20)
No	44 (80)
**Adjuvant chemotherapy**	
Yes	9 (90)
No	1 (10)
**Adjuvant radiotherapy**	
Yes	4 (40)
No	6 (60)

**Table 2:** Distribution of the first malignancy among the patients, the time interval between the first malignancy and pancreatic ductal adenocarcinoma (PDAC) diagnosis, and the association with the delta class.

First primary malignancy	N (%)	Time interval in years: Median (Range)	Delta score		
			High	Low	P value*
Lymphoma	13 (23)	9.8 (0.6–42.7)	4	9	0.06
Bladder cancer	8 (14)	1.7 (0.1–4.2)	4	4	0.8
Colorectal cancer	8 (14)	4 (1.2–13.2)	6	2	0.1
Lung cancer	6 (11)	1.6 (0.15–7.4)	3	3	0.8
Renal cancer	5 (9)	3.5(0.18–6.9)	3	2	0.7
Breast cancer	2 (4)	11.6 (2.1–21.1)	1	1	0.8
Melanoma	2 (4)	5.3 (6.1–5.3–6.9)	1	1	0.8
Endometrial cancer	2 (4)	4.2 (1.4–7.1)	2	–	0.1
Ovarian cancer	2 (4)	10.2 (9.3–11.1)	1	1	0.8
Others	7 (15)	10 (1.2–25.4)	4	3	0.8

*likelihood ratio test weight each first malignancy types versus all other types combined.

### Tumor Growth Kinetics

**Figure 1:**
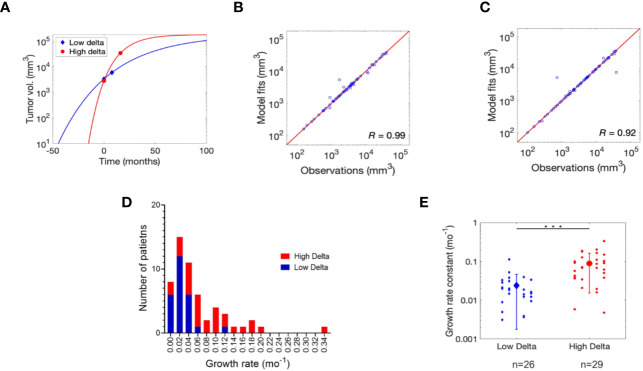
Gompertz function fitting and parameter estimation. **(A)** Non-linear least squares regression fits of Gompertz function to tumor growth kinetics data for one representative patient each bearing low delta and high delta tumor. Refer to Figure S1 for the remaining patient data fits. Pearson correlation analysis to assess quality of model fits relative to clinical observations in **(B)** low delta and **(C)** high delta tumors. **(D)** Distribution of the growth rate for high and low delta tumors. **(E)** Estimates of growth rate constant (α) of low and high delta tumors. *** *P*-value < 0.0001.

The Gompertz function accurately fits the individual patient data (**Figures 1A** and **S1**), as indicated by a strong correlation between the observed and fitted values of tumor size leading to a Pearson correlation coefficient *R* of 0.99 and 0.92 for low delta and high delta tumors, respectively (**Figures 1B, C**). The estimated growth rate constant (α) of low delta tumors was significantly lower than high delta tumors (t-test, p<0.0001), with mean value of 0.024 month^−1^ and 0.088 month^−1^, respectively (**Figures 1D, E**). These values of growth rate constants correspond to average characteristic tumor growth times of ~41 months and ~11 months for low delta and high delta, respectively.

**Figure 2:**
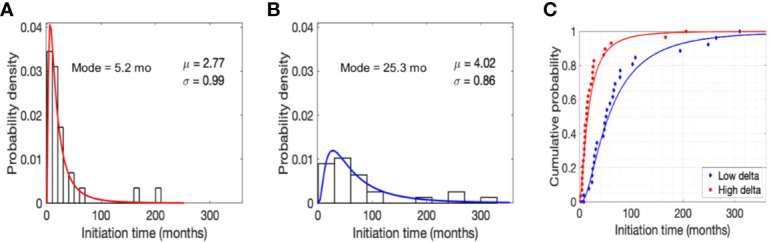
Model predictions. Normalized histogram for time to grow (initiation time) from a single cell to a tumor size of 10 mm^3^ in high **(A)** and low **(B)** delta tumors. Parameters μ and σ refer to the mean and standard deviation of lognormal distribution, respectively. Cumulative probability of initiation time in high and low delta tumors **(C)**.

As shown in **Figures 2A, B**, the data distribution for initiation times was positively skewed, hence a lognormal distribution appropriately represents the probability density of initiation times (time to grow from 1 cell to a 10 mm^3^ mass) for the two tumor types. However, in accordance with the observation above, initiation times for low delta tumors were less positively skewed than high delta tumors, with a distribution mode of 25.3 months versus 5.2 months and values ranging up to ~26 years versus ~17 years for low delta and high delta tumors, respectively (**Figures 2A, B**). Finally, we calculated that with 90% probability, the initiation time of low delta tumors was ~14 years and that of high delta tumors was ≤5 years (**Figure 2C**). These observations indicate that low delta tumors grow at a relatively slower rate than high delta tumors.

### Binary Tumor Classification

**Figure 3:**
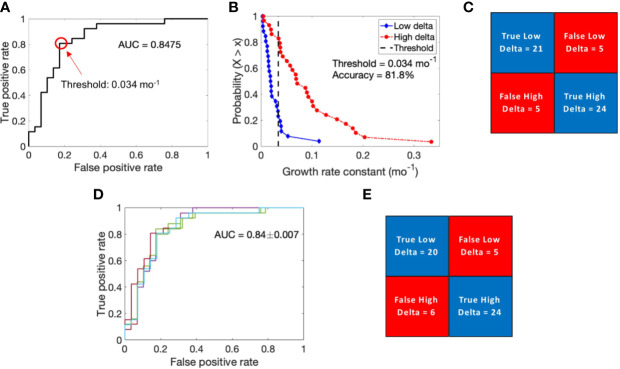
Logistic regression-based binary classification and cross-validation. **(A)** Receiver operating characteristic (ROC) curve to evaluate the classification ability of growth rate constant into low delta and high delta tumors. **(B)** Complementary cumulative distribution function (CCD) of patients shows the accuracy of binary classification at a discrimination threshold of 0.034 mo^−1^. **(C)** Confusion matrix showing results of binary classification. **(D)** ROC curves generated for multiple training data sets obtained through the leave-one-out cross validation technique. **(E)** Results of cross validation in classifying the test data point.

To further validate the hypothesis that tumor growth kinetics vary between high and low delta tumors, we performed logistic regression-based binary classification analysis to classify high and low delta tumors based on their growth rate constants (α). The obtained ROC curve had an AUC of ~0.85, which indicates good classification ability of the growth rate constant (**Figure 3A**). From the ROC curve, 0.034 month^−1^ was selected as the optimal cut-off value to differentiate the tumors into high and low delta (values >0.034 month^−1^ indicate high delta tumors, while <0.034 month^−1^ indicate low delta tumors). To visualize the binary classification based on the chosen threshold, we plotted the complementary cumulative distribution (CCD) function of the growth rate constant data. As shown in **Figures 3B, C**, the growth rate constant correctly classified ~81% and ~83% low delta and high delta tumors, respectively, with an overall accuracy of classification being ~82%. The classifier achieved high sensitivity (~83%, and ~81%), specificity (~81%, and ~83%), positive predictive value (~83%, and ~81%), and negative predictive value (~81% and ~83%) in identifying the high and low delta tumors, respectively.

The calculated value of Matthews correlation coefficient of +0.64 suggests good correlation between the predicted values and the true values of tumor type, and corroborates the classification ability of the classifier (26).

To evaluate the predictive ability of the binary classifier, we performed LOOCV. The average AUC thus obtained for the ROC curves generated by iteratively removing one data point from the training data was 0.84 ± 0.007, which was very similar to the AUC of the complete training data set (**Figure 3D**). Based on each training data, we classified the left-out test data point, pooled the results of all the iterations, and obtained an overall classification accuracy of 80%, with ~77% and ~83% of low delta and high delta tumors correctly classified, respectively (**Figure 3E**). Using LOOCV, the classifier achieved high sensitivity (~83% and ~77%), specificity (~77%, and ~83%), positive predictive value (80%, and 80%), and negative predictive value (80% and 80%) in identifying the high and low delta tumors, respectively.

### Inter- and Intra-Rater Variability Assessment

The ICC test showed excellent inter-rater agreement rates for the calculated lesion volumes on the pre-diagnostic scans (0.98, 95% CI: 0.95–0.99) and diagnostic scans (0.99, 95% CI: 0.99–0.94). Similarly, the ICC model showed excellent intra-rater agreement rates for the calculated lesion volumes on the pre-diagnostic scans (0.99, 95% CI: 0.98–0.99) and diagnostic scans (0.99, 95% CI: 0.99–0.1) (**Supplementary Table 3**).

### Association Between Delta Score, Soft Tissue Wasting, and BG

**Figure 4:**
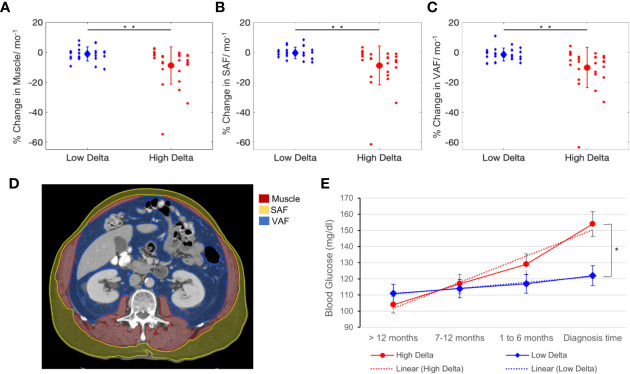
Soft tissue and metabolic analysis. Rate of change of tissue wasting in muscle **(A)**, subcutaneous abdominal fat (SAF) **(B)**, and visceral abdominal fat (VAF) **(C)** in patients with high and low delta tumors. **(D)** Muscle, SAF, VAF contours on CT-scans at L2 vertebra level. **(E)** Blood glucose kinetics in high and low delta tumor-bearing patients. T-test *p value < 0.01, **p value < 0.001.

A significant difference was observed between the rates of soft tissue wasting of high and low delta tumors, as confirmed by t-tests, such that patients with high delta tumors experienced accelerated rate of subcutaneous fat (−8.7 vs. −1.1% change/month, p < 0.001), visceral fat (−10.2 vs. −1.5% change/month, p < 0.001), and muscle (−8.8 vs. −0.4% change/month, p < 0.001) wasting compared to those with low delta tumors (**Figures 4A–D**). Additionally, there was a significant difference in the temporal profile of the patients’ BG, whereby those with high delta tumors exhibited a higher increase in the BG in the pre-diagnostic period compared to those with low delta tumors (p = 0.004) (**Figure 4E**).

Since the diagnosis age of PDAC in our cohort had a relatively wide range, we tested whether the difference in the basal metabolic rates across age groups was a confounding factor to consider. We dichotomized the study subjects based on the median age (68 years). The t-test did not show any significant difference in the rate of subcutaneous fat (p=0.9), visceral fat (p=0.07), and muscle (p=0.8) wasting between the age groups.

### Association Between Delta Score and Clinical Outcomes

**Figure 5:**
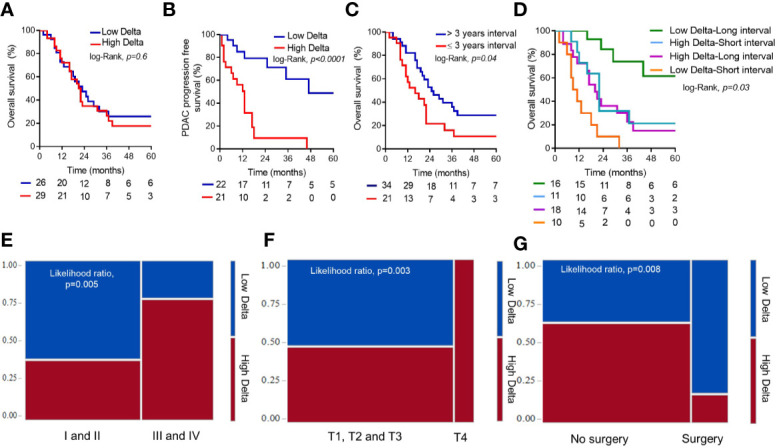
Survival analysis. Delta score association with overall survival **(A)** and progression free survival **(B)**. Comparison of the time interval between the first and second primary and overall survival **(C)**. Delta score and time interval association with overall survival **(D)**. Contingency plots showing delta score associations with overall stage at pancreatic ductal adenocarcinoma (PDAC) diagnosis **(E)**, T-stage at PDAC diagnosis **(F)**, and with the likelihood to receive a curative intent PDAC resection **(G)**.

There was no significant association between delta score and OS (log-rank, p = 0.6) (**Figure 5A**). However, patients with low delta tumors demonstrated improved PDAC-specific PFS (47 vs. 6 months, Log-Rank p < 0.0001), presented with earlier overall stage of disease, were more likely to have T1-T3 stage tumors, and were more likely to receive surgical resection (likelihood ratio, p = 0.008), compared to those with high delta tumors (**Figures 5B–G**).

As a continuous variable, patients with a longer time interval between the first and second primary experienced better OS (HR = 0.94, 95%CI = 0.8–0.9, p = 0.01). Using ROC curve analysis, 3 year interval was selected as an optimal cut-off (AUC=0.71) to best predict prolonged OS (>22 months), and was used to dichotomize the patients into long interval vs. short interval groups. As a categorical variable, this dichotomy showed a significant association with OS (32 vs. 20 months, Log-Rank; p = 0.03) (**Figure 5C**). We combined the delta score and the time interval to create four groups; 1) high delta-short interval, 2) high delta-long interval, 3) low delta-short interval, and 4) low delta-long interval. The low delta-long interval group had significantly better OS compared to other groups (37 vs. 22 vs. 20 vs. 10 months, log-rank p = 0.0005) (**Figure 5D**).

Multivariate Cox proportional-hazards analysis, showed that time interval between the primary and secondary cancer was the only independent prognostic factor for survival accounting for traditional covariates (HR = 0.9, p = 0.04), as shown in **Table 3**.

**Table 3:** Univariate and multivariate Cox proportional hazard analysis for overall survival.

Characteristic	No. of patients	Univariate Analysis		Multivariate Analysis	
		HR (95%CI)	P value	HR (95%CI)	P value
**Delta (†)**					
High	29	1.1(0.6-2.1)	0.6	–	–
Low **Growth rate (α)**	26 55	- 3.6(0.7–16.1)	- 0.11	- -	- -
**Surgery**					
Yes	10	0.3(0.13–0.9)	*0.02*	0.5 (0.16–1.5)	0.2
No	45	–	–	–	–
**Age (years)**	55	1(0.9–1.05)	0.3	–	–
**Sex**					
Female	22	0.9 (0.49–1.7)	0.8	–	–
Male	33	–		–	–
**Stage**					
I and II	34	0.6(0.3–1.2)	0.21	–	–
III and IV	21				
**Chemo/radiation therapy**					
Yes	43	1.6(0.82–3.9)	0.15	1.2(0.5–3.2)	0.6
No	12				
**Years between 1^st^ and 2^nd^ cancer**	55	0.94(0.8–0.99)	*0.01*	0.95(0.9–0.99)	*0.04*
**Delta (††)**				*	*
High	2	0.9 (0.02–6.2)	0.9	–	–
Low **Growth rate (α)**	8 10	- 1.5(0.03–22.8)	- 0.7	- -	- -
**Surgical margin**					
Positive (R1)	1	3.5 (1.5–15)	*0.03*	–	–
Negative (R0)	9	–	–	–	–
**N Stage**					
Positive (N1)	4	11 (1.6–230)	*0.01*	–	–
Negative (N0)	6	–	–	–	–
**Adjuvant chemo/radiation**					
Yes	9	0.2 (0.02–5.7)	0.3	–	–
No	1				

^(†)^All patients who developed pancreatic cancer as a second primary (n = 55).

^(††)^Patients who underwent surgical resection (n = 10).

*No sufficient events to run multivariate analysis (data overfitting).

## Discussion

In this paper, we utilized phenomenological mathematical modeling to characterize the growth kinetics of imaging-based subtypes of PDAC, in addition to evaluating the differential metabolic effects of these subtypes. We measured pancreatic lesions from the pre-diagnostic and diagnostic CT-scans of patients who developed PDAC as a second primary. The model identified significant differences in the growth rate constant and initiation time between the subtypes, whereby high delta tumors exhibited an accelerated growth rate and shorter initiation time compared to low delta tumors. Moreover, patients with high delta tumors exhibited greater metabolic profile disturbances in terms of soft tissue wasting and hyperglycemia. The patients with high delta tumors were more likely to present with advanced stage disease and had poorer clinical outcomes compared to those with low delta tumors. These findings provide additional insights into the biological, metabolic and clinical aspects of these subtypes and suggest that screening strategies may require personalization, factoring biology into the intervals at which patients undergo imaging.

For example, our data illustrated that patients with high delta tumors have shorter doubling times (calculated from the tumor growth rate, mean=4.2 ± 3 months) compared to those with low delta tumors (mean=16 ± 8 months). Indeed, patients with high delta tumors presented with more advanced T- and overall stages of the disease. These results suggest that the one-size-fits-all screening approach is inadequate. Current protocols use a one-year screening interval, but this would potentially miss an early stage high delta tumor. This emphasizes the need for personalized approaches to screening for PDAC, but also highlights an unmet need: there is currently no method to predict whether a high-risk patient undergoing screening will develop no disease, indolent PDAC, or aggressive PDAC.

Secondary signs that are associated with development of PDAC that can be measured in blood or imaging may help address this unmet need. Multiple studies investigated the association between the development of pancreatic cancer and the onset of metabolic changes in terms of soft tissue wasting and blood chemistry disturbance (8–10). Sah et al. demonstrated that there are three distinct phases of soft tissue (fat and muscle) wasting, hyperglycemia, and dyslipidemia that precedes the diagnosis of PDAC (8). Our serial quantitative analyses of fat and muscle changes on CT-scans and BG level disturbances were consistent with these findings. Moreover, we found significant differences in the rates of these changes between high and low delta tumors, further supporting that these imaging-based subtypes are biologically and metabolically different. Additionally, the association between these measurable changes and the imaging-based subtypes provides a potential solution to personalizing screening intervals for high risk cohorts.

Characterizing the tumor growth pattern is of diagnostic and prognostic relevance. Haeno et al. used mathematical modeling and clinical data to illustrate that controlling the growth rate of PDAC, especially at the early exponential phase, is more effective for prolonging patients’ survival than surgical resection (27). Since we previously observed multiple differences in the biology of high and low delta tumors (20, 28), we hypothesized that the growth pattern and proliferation kinetics of these tumor subtypes would be fundamentally different. Our finding that the delta subtypes have differential growth rates aligns with our earlier observation regarding the morphological characteristics of the delta as a function of opposing proliferation versus migration mechanisms of tumors cells (19).

This study has a few limitations. First, due to the retrospective nature of the study, the time intervals between pre-diagnostic and diagnostic CT-scans were not uniform across all patients. Notably, however, intervals were not significantly different between high and low delta cases (mean = 7.3 months vs 8.3 months, respectively). Also, the imaging protocols of the pre-diagnostic (T0) CT-scans varied from single phase (PV) scans to triple phase scans with and without contrast. This is explained by the variability in the location, stage and the indication for imaging of the first malignancy, i.e. surveillance (n=29) versus management and follow-up (n=26). However, there was at least one contrast enhanced CT-scan at T0 that was used for tumor measurement. Second, with our mathematical model, we assumed that PDAC tumors consist mainly of cancerous cells that originate from a single mutated cell, which might not be the case for all the tumors. While the Gompertz function has been successfully used to describe tumor growth previously (29, 30), it assumes that the tumor growth is fastest early on and slows down with time. While this appears to capture the reported PDAC growth pattern (27), this remains an approximation. Our future work will address both multi-cell origin and consider inter- and intra- tumor heterogeneity with an aim to also understand tumor metastasis. Another potential weakness of our work is that we used biaxial measurements to estimate the 3D tumor volume instead of the precise tumor volume. Finally, we acknowledge that the data was from a single institution with limited number of patients that requires further external validation. Future directions include multi-institutional validation, developing a deep learning-based technique to detect and classify the imaging-based subtypes of PDAC, investigating the molecular basis associated with different growth patterns, and enhancing CT imaging capacities to detect PDAC earlier through amplifying faint abnormal signals in the pancreas (31).

In conclusion, we used mathematical modeling to characterize the growth rates and proliferation kinetics of imaging-based subtypes of PDAC, using serial pre-diagnostic CT scans of patients who developed PDAC as a second primary. We highlighted the biological and metabolic differences associated with these subtypes. With further validation, these findings have implications for personalized screening strategies for PDAC.

## Data Availability Statement

The raw data supporting the conclusions of this article will be made available by the authors, without undue reservation.

## Ethics Statements

The studies involving human participants were reviewed and approved by Institutional Review Board of MD Anderson Cancer Center. Written informed consent for participation was not required for this study in accordance with the national legislation and the institutional requirements.

## Author Contributions

All authors listed have made a substantial, direct and intellectual contribution to the work, and approved it for publication.

## Funding

We gratefully acknowledge support from the Andrew Sabin Family Fellowship, the Sheikh Ahmed Center for Pancreatic Cancer Research, institutional funds from The University of Texas MD Anderson Cancer Center, the Khalifa Foundation, equipment support by GE Healthcare and the Center of Advanced Biomedical Imaging, Philips Healthcare, and Cancer Center Support (Core) Grant CA016672 from the National Cancer Institute to MD Anderson. Dr. Eugene Koay was supported by NIH (U54CA210181, U54CA143837, U01CA200468, U01CA196403, U01CA214263, R01CA221971, R01CA248917, and R01CA218004) and the Pancreatic Cancer Action Network (16-65-SING).

## Conflict of Interest

BW revives grant support form Celgene and Eli Lilly, and is consulting for BioLineRx, Celgene, and GRAIL.

The remaining authors declare that the research was conducted in the absence of any commercial or financial relationships that could be construed as a potential conflict of interest.

The reviewer SM declared a past co-authorship with several of the authors, AG and DV, to the handling editor.

## Supplementary Material

Supplementary Figure 1 Gompertz function fitting. Non-linear least squares regression fits of Gompertz function to tumor growth kinetics data for **(A)** low delta and **(B)** high delta tumors. Markers (circles and triangles) represent raw data and solid lines represent model fits.
