# Supplementary figures and images for: Imaging-Based Subtypes of Pancreatic Ductal Adenocarcinoma Exhibit Differential Growth and Metabolic Patterns in the Pre-Diagnostic Period: Implications for Early Detection
Source: Front Oncol. 2020 Dec 2;10:596931. doi: 10.3389/fonc.2020.596931 (PMC7738633; doi:10.3389/fonc.2020.596931)

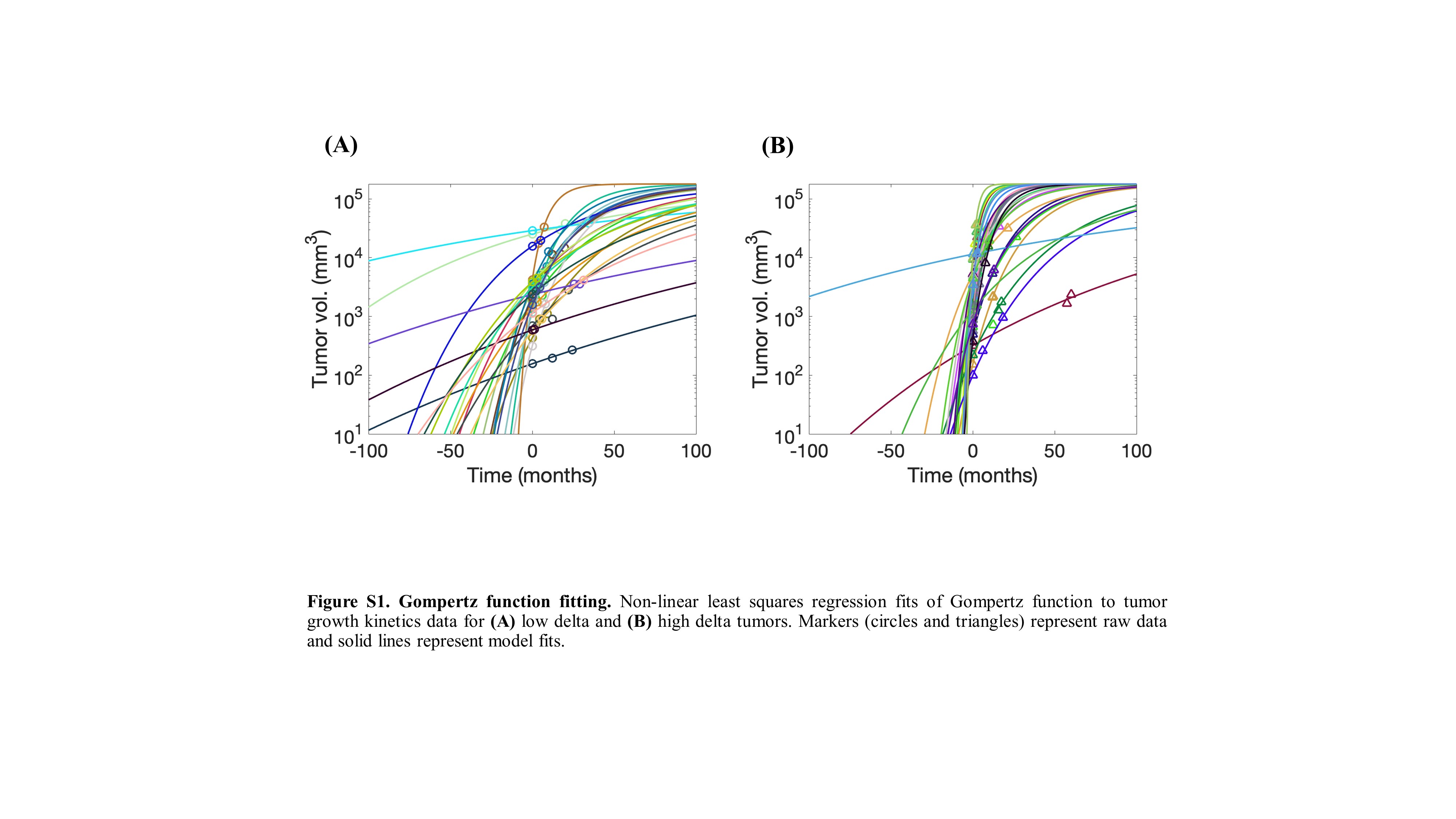

Supplement: Supplementary Figure 1 — Gompertz function fitting. Non-linear least squares regression fits of Gompertz function to tumor growth kinetics data for (A) low delta and (B) high delta tumors. Markers (circles and triangles) represent raw data and solid lines represent model fits. [file Image_1.jpeg]
