# Supplementary material for: Imaging-Based Subtypes of Pancreatic Ductal Adenocarcinoma Exhibit Differential Growth and Metabolic Patterns in the Pre-Diagnostic Period: Implications for Early Detection
Source: Front Oncol. 2020 Dec 2;10:596931. doi: 10.3389/fonc.2020.596931 (PMC7738633; doi:10.3389/fonc.2020.596931)
Supplement: Supplementary file 2 [file Table_1.docx]

| **Characteristic** | **N (%)** |
| --- | --- |
| **Number of pre-diagnostic CT-scans** |  |
| 1 | 44 (80) |
| 2 | 7 (13) |
| 3 | 3 (5) |
| 4 | 1 (2) |
| **Time interval (months)**  Between T0 and T1 (median, range) | 5.7 (1 - 57) |
| **Supplementary table S1:** Number of pre-diagnostic CT scans and the interval between T0 (the time point when a pancreatic lesion was spotted) and T1 (the time point when pancreatic cancer was diagnosed) | |
|  | |
