# Supplementary material for: Imaging-Based Subtypes of Pancreatic Ductal Adenocarcinoma Exhibit Differential Growth and Metabolic Patterns in the Pre-Diagnostic Period: Implications for Early Detection
Source: Front Oncol. 2020 Dec 2;10:596931. doi: 10.3389/fonc.2020.596931 (PMC7738633; doi:10.3389/fonc.2020.596931)
Supplement: Supplementary file 3 [file Table_2.docx]

| **First primary malignancy** | **Chemo/radiation therapy regimen  (No. of patients)** | **Surgery (No.)** | **Delta Score** | |
| --- | --- | --- | --- | --- |
|  |  |  | **High** | **Low** |
| Lymphomas | CHOP + Rituxmab +/- radiation (8)  EPOCH + Rituxmab +/- radiation (2)  MOPP + Radiation (1)  Rituxmab + Bendamustine (1)  Radiation (1) | NA | 2  1  -  -  1 | 6  1  1  1  - |
| Bladder cancer | Gemcitabine based chemotherapy^*^ (4)  MVAC (2)  BCG immunotherapy (1)  None (1) | 4 | 1  1  1  1 | 3  1  -  - |
| Colon cancer | Capecitabine +/- radiation (2)  FOLFOX (2)  None (4) | 8 | -  2  4 | 2  -  - |
| Renal cancer | Gemcitabine + Carboplatin + Bevacizumab (1)  None (4) | 5 | -  3 | 1  1 |
| Lung cancer | Carboplatin based ^**^ +/- radiation (3)  Radiation (2)  Cisplatin, Docetaxel +/- radiation (1) | 3 | 2  1  - | 1  1  1 |
| Endometrial cancer | Carboplatin, paclitaxel + radiation (1)  Radiation (1) | 2 | 1  1 | -  - |
| Ovarian cancer | Carboplatin + Paclitaxel (2) | 2 | 1 | 1 |
| Breast cancer | Docetaxel + radiation (1)  Radiation (1) | 2 | 1 | 1 |
| Melanoma | Radiation (1)  None (1) | 2 | 1 | 1 |
| Others | Radiation (2)  Radioactive Iodine (1)  None (2)  Dasatinib (1)  Thalomid + Decadron (1) | 3 | 1  -  1  1  1 | 1  1  1  -  - |
| **Supplementary table S2:** Treatment regimen and surgery status of the first malignancy and distribution of the associated delta score of the secondary PDAC  CHOP : Cyclophosphamide, Doxorubicin, Vincristine, and Prednisone  EPOCH: Etoposide, Prednisone, Vincristine, and Cyclophosphamide and Doxorubicin  MOPP: Chlormethine, Vincristine, Procarbazine and Prednisone  MVAC: Methotrexate, Vinblastine, Doxorubicin and Cisplatin  ^*^Gemcitabine based include combinations with Cisplatin, Ifosfamide, and/or Paclitaxel  ^**^Carboplatin based include Paclitaxel, Bevacizumab and/or Nivolumb | | | | |
