# Supplementary material for: Imaging-Based Subtypes of Pancreatic Ductal Adenocarcinoma Exhibit Differential Growth and Metabolic Patterns in the Pre-Diagnostic Period: Implications for Early Detection
Source: Front Oncol. 2020 Dec 2;10:596931. doi: 10.3389/fonc.2020.596931 (PMC7738633; doi:10.3389/fonc.2020.596931)
Supplement: Supplementary file 4 [file Table_3.docx]

|  | **Variable** | | **Average** | **Lower 95%CI** | **Upper 95%CI** | **Agreement interpretation** |
| --- | --- | --- | --- | --- | --- | --- |
| **Pre-diagnostic volumes ICC** | Intra-rater | All | 0.994 | 0.984 | 0.998 | Excellent |
|  |  | High delta | 1.000 | 0.998 | 1.000 | Excellent |
|  |  | Low delta | 0.994 | 0.969 | 0.999 | Excellent |
|  | Inter-rater | All | 0.983 | 0.952 | 0.994 | Excellent |
|  |  | High delta | 0.988 | 0.94 | 0.997 | Excellent |
|  |  | Low delta | 0.982 | 0.913 | 0.996 | Excellent |
| **Diagnostic volumes ICC** | Intra-rater | All | 0.999 | 0.998 | 1.000 | Excellent |
|  |  | High delta | 1.000 | 0.999 | 1.000 | Excellent |
|  |  | Low delta | 0.999 | 0.995 | 1.000 | Excellent |
|  | Inter-rater | All | 0.998 | 0.994 | 0.999 | Excellent |
|  |  | High delta | 0.999 | 0.996 | 1.000 | Excellent |
|  |  | Low delta | 0.997 | 0.982 | 0.999 | Excellent |
| **Supplementary Table (3) Intra- and inter-rater agreement of Intraclass correlation coefficient (ICC) in 16 patients** | | | | | | |
